# The impact of the open-access status on journal indices: a review of medical journals

**DOI:** 10.12688/f1000research.17979.1

**Published:** 2019-03-07

**Authors:** Saif Aldeen AlRyalat, Mohammad Saleh, Mohammad Alaqraa, Alaa Alfukaha, Yara Alkayed, Maryann Abaza, Hadeel Abu Saa, Mohamed Alshamiry

**Affiliations:** 1 Department of Ophthalmology, University of Jordan, Amman, 11942, Jordan; 2 School of Medicine, University of Jordan, Amman, 11942, Jordan

**Keywords:** Open access, Journal, Medicine, Bibliometrics, Citation

## Abstract

**Background:** Over the past few decades, there has been an increase in the number of open access (OA) journals in almost all disciplines. This increase in OA journals was accompanied an increase in funding to support such movements. Medical fields are among the highest funded fields, which further promoted its journals to move toward OA publishing. Here, we aim to compare OA and non-OA journals in terms of citation metrics and other indices.

**Methods:** We collected data on the included journals from Scopus Source List on 1 ^st^ November 2018.  We filtered the list for medical journals only. For each journal, we extracted data regarding citation metrics, scholarly output, and wither the journal is OA or non-OA.

**Results:** On the 2017 Scopus list of journals, there was 5835 medical journals. Upon analyzing the difference between medical OA and non-OA journals, we found that OA journals had a significantly higher CiteScore (p< 0.001), percent cited (p< 0.001), and source normalized impact per paper (SNIP) (p< 0.001), whereas non-OA journals had higher scholarly output (p< 0.001). Among the five largest journal publishers, Springer Nature published the highest frequency of OA articles (31.5%), while Wiley-Blackwell had the lowest frequency among its medical journals (4.4%).

**Conclusion:** Among medical journals, although non-OA journals still have higher output in terms of articles per year, OA journals have higher citation metrics.

## Introduction

Open access (OA) journals allow free (access to/availability of) academic articles, they enable any user to read, search, download, share, use them for indexing, print the full texts, or utilize them as data for software without being charged ^1^. Over the past 20 years, there has been an increase in the number of OA medical journals. According to Web of Science, published OA articles as a proportion of total publications increased from 9.5% to 24% from 1998 to 2018. These OA journals provide an easily accessed source of information, a source that is accessible even for developing and low income countries ^2^.

Bibliometric analysis are methods or applications used to measure the influence of authors or scientific papers, of which, citation analysis is the most commonly used methods ^3^. Now several citation databases have become available, with the three largest being Web of Science, Scopus and PubMed. These databases record the number of times that a journal article has been cited by other papers ^4^. The use of bibliometric analysis is becoming more popular to assess the performance of different aspects of the scholarly and scientific fields. Analysis can be at the level of the researchers themselves, journals, departments, universities, national organizations, and even entire nations ^5– 8^. There are several databases that can be used to perform the bibliometric analysis, with each database having its own characteristics; these include Google Scholar, Pubmed (Only biomedical citations), Scopus, and Web of Science ^4^. According to the number, coverage, and quality of citations covered by the databases, Scopus has wide coverage of high quality journals, compared to high number of citations at the expense of quality for Google Scholar, and high quality at the expense of number of citations for Web of Science ^9– 11^.

It is claimed that the emergence of OA journals has led to better dissemination of knowledge with the additional benefit of more citations for the authors, although this is still a matter of debate ^12^. In this study, we aim to study the OA status of medical journals and the impact of the open-access status on journal indices using the Scopus database.

## Methods

### Data collection

We collected data on the included journals from Scopus Source List on 1 ^st^ November 2018 (see Underlying data ^13^). We filtered the list for medical journals (which include all specialties in medicine, as per Scopus categorization).

### Variables

For each journal, we extracted the following citation metrics: Citation count, Percent Cited, CiteScore, CiteScore Percentile, SCImago Journal Rank, Source Normalized Impact per Paper (SNIP), and SCImago Quartiles. Details about these metrics and how they are calculated can be found on Scopus website.

Moreover, scholarly output is defined as sum of documents published in the serial title (e.g. 2017) in the 3 years prior to the year of the metric (e.g. 2014 – 16). Open access Journals covered by Scopus are indicated as Open Access if the journal is listed in the Directory of Open Access Journals (DOAJ) and/or the Directory of Open Access Scholarly Resources (ROAD).

### Statistical analysis

We used SPSS version 22.0 (Chicago, USA) in our analysis. We used means (± standard deviation) to describe continuous variables (i.e. journal indices). We used counts (frequency) to describe other nominal variables (i.e. publishers and OA journals). We performed Mann-Whitney tests to analyze the difference between measurements and OA status, and we presented data as medians (25% to 75% quartiles). To analyze open access journals between radiology and medicine, we used the weighting cases function in SPSS and a Chi-square test. All underlying assumptions were met, unless otherwise indicated. A p value of 0.05 was considered as significant.

## Results

In the 2017 Scopus list of journals, there was 5835 medical journals. Regarding the 5 most common publishers, 890 (15.3%) journals were from Elsevier, 653 (11.2%) Springer Nature, 196 (6.8%) Taylor & Francis, 360 (6.2%) Wiley-Blackwell, and 304 (5.2%) Wolters Kluwer. 1293 (22.2%) journals were OA journals. Table 1 indicates the minimum, maximum, mean, and standard deviation of medical journal indices.

**Table 1.** Descriptive statistics for medical journals.

	N	Minimum	Maximum	Mean	Std. Deviation
CiteScore	5835	0	130	1.58	2.588
Percentile	5835	0	99	48.03	28.666
Citation count	5835	0	77809	761.87	2221.841
Scholarly output	5835	1	11270	346.14	505.062
Percent cited	5835	0	100	45.67	26.800
SNIP	5835	0.000	88.164	0.75260	1.450990
SJR	5835	0.000	61.786	0.82674	1.572423
Rank	5835	1.00	785.00	162.7102	167.95247

SNIP: Source Normalized Impact per Paper; SJR: SCImago Journal Rank.

Upon analyzing the difference between medical OA and non-OA journals, we found significant differences in the following indices:

- CiteScore (p< 0.001): with a median of 1.19 (25–75%: 0.53–2.21) for OA journals, and a median of 1.06 (25–75%: 0.26–2.18) for non-OA journals.
- Scholarly output (p< 0.001): with a median of 157 (25–75%: 76–319.5) for OA, and a median of 205 (25–75%: 107–423) for non-OA journals.
- Percent cited (p< 0.001): with a median of 52% (25–75%: 32%-70%) for OA, and a median of 48% (25–75%: 19%–68%) for non-OA journals.
- SNIP (p< 0.001): with a median of 0.706 (25–75%: 0.370–1.023) for OA, and a median of 0.617 (25–75%: 0.176–1.013) for non-OA journals.

Upon comparing open access journals between the 5 most common publishers, we found a significant difference (p< 0.001). Post-hoc analysis showed that Wiley-Blackwell has significantly lower number of open access journals 16 (4.4%) open access journals compared to others. Table 2 shows the open access status for the most common publishers.

**Table 2.** A comparison in the percentage of open access (OA) journals between the top five publishers of medical journals.

			Open access		Total
			No	Yes	
publishers	Elsevier	Count	756	134	890
			84.9%	15.1%	100.0%
	Springer Nature	Count	447	206	653
			68.5%	31.5%	100.0%
	Taylor & Francis	Count	324	72	396
			81.8%	18.2%	100.0%
	Wiley-Blackwell	Count	344	16	360
			95.6%	4.4%	100.0%
	Wolters Kluwer Health	Count	229	75	304
			75.3%	24.7%	100.0%
	Others	Count	2442	790	3232
			75.6%	24.4%	100.0%
Total		Count	4542	1293	5835
			77.8%	22.2%	100.0%

## Discussion

Our study found that OA medical journals had significantly higher CiteScores, Percent cited and SNIP; which is consistent with a number of previous studies made across a variety of disciplines including philosophy, political science, engineering, mathematics, physics, computer science and agriculture; all of which concluded that open access publications have a greater research impact (higher citation rate) than non-open access publications ^12, 14– 16^. On the other hand, in a randomized controlled trial conducted on 11 biological and medical journals, it was found that only 2 of these journals showed positive and significant OA effects. In addition, it was found that OA advantage is declining by about 7% per year, from 32% in 2004 to 11% in 2007 ^17^. Chua *et al*. found that there was significantly more citations in OA articles than in non-OA articles within almost identical journals’ impact factor ^18^. Moreover, comparing citations in OA and non-OA articles in the same journal showed significant citation privilege for OA publications in several studies. For example, for the Journal of Postgraduate Medicine a comparison of citations per 100 articles per year before and after the journal became open access showed an increase between 3 and 4.5 times in citations ^19^. In a longitudinal study of a cohort of OA and non-OA articles, it was shown that OA articles are cited earlier, and almost 2 times more frequently than non-OA articles in the first 4–16 months after publication in the same journal ^20^. Regardless of all the aforementioned findings, our study found that non-OA medical journals have significantly higher Scholarly Output which can be strongly linked to the fact that most non-OA medical journals have been established years before OA journals, which have only recently emerged ^21^.

We found that the number of OA journals varied among publishers, with Whiley-Blackwell having the least, with only 16 journals (4.4%), and the most with Springer Nature (206, 31.5%). In a previous study that analyzed OA articles published by different publishers, regardless of the discipline, they found that Elsevier had the highest number of OA articles, followed by Springer Nature and Whiley-Blackwell ^22^. A longitudinal study comparing hybrid open access articles between publishers found great variation depending on the discipline ^23^. For instance, medicine is the discipline which most frequently publishes in hybrid OA ^23^.

Our study has potential limitations. In this study, we didn’t account for the effect of publishing OA articles in non-OA journals (hybrid journals), as “Gold” OA publishing (i.e. fully OA journals) relates to publication of articles that are freely available to view and these may occur in OA or hybrid journals. Moreover, future studies should consider analyzing specialties within medicine (e.g. oncology), where we believe there will be variations in the effect of OA publishing within these specialties.

## Data availability

### Underlying data

Harvard Dataverse: Medical journals. https://doi.org/10.7910/DVN/YYUTGG ^13^.

This project contains the following underlying data:

- Medical journals 2017 dataset.tab (Scopus search results from the 1 ^st^ November 2018)

Data are available under the terms of the Creative Commons Zero "No rights reserved" data waiver (CC0 1.0 Public domain dedication).
