# Supplementary material for: The impact of the open-access status on journal indices: a review of medical journals
Source: F1000Res. 2019 Mar 7;8:266. [Version 1] doi: 10.12688/f1000research.17979.1 (PMC6449789; doi:10.12688/f1000research.17979.1)
Supplement: Peer review history

# Peer review: Reviewer response for version 1

**DOI:** 10.5256/f1000research.19663.r45999

**Published:** 2019-04-03

**Authors:** Sung-Tae Hong

**Affiliations:** 1 Seoul National University College of Medicine, Seoul, South Korea

General comment: This article describes difference of journal citation metrics between OA and non-OA journals in medical scope. The subjected journals are 5835 medical journals which are indexed in the Scopus and compared indices are metrics data supplied by Scopus and SCImago. The data showed significantly higher citation indices in OA journals than those in non-OA journals. The conclusion is that OA medical journals are cited more. The conclusion is known already in OA journals of other disciplines but it is valuable to publish.

 The conclusion is sound and positive as expected, but I think we have to think about other issues on this topic. OA means no financial barrier for readers, which can facilitate citations. But most of researchers in established institutions have no barrier to non-OA journals because their institutions are able to subscribe to the non-OA journals. For those researchers, non-OA journals are free. I think that is the main reason of the small difference of those indices between the two groups although the difference is statistically significant. The statistics must be supported by large number of subject journals. This point must be discussed.

 Specific comments

1. Definition of OA: journals included in DOAJ or ROAD. There are more Scopus journals outside of the 2 directories. PubMed Central may add more OA journals.
2. The core results are difference of the indices that must be presented in a Table. Tables 1 and 2 are supportive but not cores. For reader friendly writing, one Table is required for results described in the text.
3. Table 2 analyzed total numbers of OA journals by publishers. It may be interesting for readers to summarize whether there are index differences by publishers.
4. Add discussion on the difference of indices: CiteScore 0.19 vs. 1.06; Scholarly output median 157 vs. 205; Percent cited 52% vs. 48%; SNIP median 0.706 vs. 0.617. All are highly significant by p<0.001. The gaps are significant but not so big because there are many other factors of citation. We should approach more details of citation analysis. 

I have read this submission. I believe that I have an appropriate level of expertise to confirm that it is of an acceptable scientific standard.

# Peer review: Reviewer response for version 1

**DOI:** 10.5256/f1000research.19663.r46001

**Published:** 2019-04-03

**Authors:** Deeb N. Salem

**Affiliations:** 1 Department of Medicine, Tufts Medical Center, Boston, MA, USA

1. The article is generally well done with respect to its method and purpose. My major concern is its purpose: assuming adequate methods, statistical analyses and result, what is the significance?  If there are implications of the research or significance of the conclusions, they should be clearly stated.
2. The article is dependent upon the accuracy and scope of the Scopus database. I wonder how that was chosen and whether Pub Med might have been consulted as well. The accuracy of the conclusions is no better than the comprehensiveness of Scorpus.
3. Are articles that appear in print but are archived online included?
4. Otherwise the study seems to have been conducted well (methodologically) and the results competently reported.

I have read this submission. I believe that I have an appropriate level of expertise to confirm that it is of an acceptable scientific standard.

# Peer review: Reviewer response for version 1

**DOI:** 10.5256/f1000research.19663.r45458

**Published:** 2019-03-19

**Authors:** Suneet Sood

**Affiliations:** 1 Jeffrey Cheah School of Medicine and Health Sciences, Monash University Malaysia, Johor Bahru, Malaysia

This paper intends to measure journal indices for OA journals, and compare them with the indices of Non-OA journals. The authors used the Scopus database.

 Although the theme is not new, I believe that the large number of indices calculated by the authors is a useful contribution to the literature.

**Database:** I am a little ambivalent about the Scopus database. It is an extensive one, but to my mind it has two characteristics that need to be kept in mind. First, Google Scholar has, in all probability, overtaken Scopus in the breadth of coverage of publications for indexing (though till about 8 years ago Google Scholar was inferior to Scopus). Secondly, GS is more lax in its selection of journals to index, and it is more than likely that several “poor quality” journals are included. The authors themselves make these observations. I submit that the so-called poor quality of the journals indexed by GS is not really a drawback, and inclusion of these journals is more likely to provide a realistic picture. That said, using the Scopus database is an acceptable choice, and the sampling is large.

**Statistics:** The choice of statistical tests is acceptable. I think that choosing two groups (OA vs Non-OA) is adequate for the objective of this paper. Would a third group, hybrid, have helped? Possibly, but hybrid journals are extremely varied in the number of articles they allow for open access, and I doubt that one hybrid journal, allowing 10% of its articles for free access, can be compared with another that allows 25% access immediately and full access after 6 months. However, I was not able to duplicate the calculations of the authors. The authors state as follows:

 CiteScore median = 1.19 for OA journals, 1.06 for non-OA journals.

 However, I calculate 1.2750 and 1.16 respectively, though the differences are still statistically significant.

 I get different values for the other metrics as well. Perhaps the authors should consider rechecking the values with their statisticians.

**Methods:** Although the methods are described with reasonable clarity, I was unsure of the period covered by the data collection. The data file suggests that the data was collected for journal issues published from 30 April 2018 onwards. Have I understood correctly? I would request the authors to provide this detail in the methods.

**Results:** The authors have recorded data for SJR values but have not discussed the results. There may be no significant differences between the two groups (OA, NOA), but I believe that they must discuss the implication of this result. The median SJR for NOA papers is slightly (but not significantly) higher than that for OA papers. Is this because NOA papers have a higher “prestige”? (Compare with their comments for scholarly output.)

**Recommendation:** The authors should consider: A. cross-checking the results, B. clarifying the time span that is covered by the study, and C. commenting on the SJR results.

I have read this submission. I believe that I have an appropriate level of expertise to confirm that it is of an acceptable scientific standard.

# Peer review: Reviewer response for version 1

**DOI:** 10.5256/f1000research.19663.r45461

**Published:** 2019-03-12

**Authors:** Ernesto Roldan-Valadez

**Affiliations:** 1 Directorate of Research, Hospital General de Mexico “Dr Eduardo Liceaga”, Mexico City, Mexico

The authors have performed an interesting study comparing differences in selected metrics (CiteScore, percent cited, SNIP, scholarly output) between medical OA and non-OA journals.

 This study, however, presents some limitations that require significant revisions:

  

- Authors are using a robust number of journals (5835 medical journals) that will allow the performance of parametric tests. I would like to see that they applied at least an ANOVA comparing three groups: OA, non-OA and OA articles in non-OA journals (hybrid journals). The hybrid group is fundamental, and they do not include it. The authors should mention to the reader the bias of this decision.
- Also in this point, it is essential the authors explain why, if previous studies have concluded that the Eigenfactorscore is the best predictor of citations ^1^^,^^2^, they did not include this metric in their analyses? Please also explain to the readers, why a linear-mixed-model design analysis was not performed.
- It is necessary to mention references of recent articles ^3^ citing the existing correlations between the selected bibliometrics (CiteScore vs SNIP, Citescore vs IF, etc.) with at least two purposes: that the authors justified why they did not include a correlation analysis in their study, and that the readers be aware of the limitations in the correlation analysis, and also how the medical speciality may influence the results. 
- It would be desirable to present a subgroup analysis of the medical specialities with the higher number of citations (for example oncology) as an example of the expected variability within subspecialties.
- If you report in the methods section that you used the SCImago Quartiles, why not control the effect of this variable using ANCOVA O MANCOVA? For example, if the authors are using the data from 5835 medical journals, this data allows a more robust analysis besides descriptive statistics and Mann-Whitney tests.

I have read this submission. I believe that I have an appropriate level of expertise to confirm that it is of an acceptable scientific standard, however I have significant reservations, as outlined above.

## References

[1] Roldan-ValadezEOrbe-ArteagaURiosC: Eigenfactor score and alternative bibliometrics surpass the impact factor in a 2-years ahead annual-citation calculation: a linear mixed design model analysis of Radiology, Nuclear Medicine and Medical Imaging journals. *Radiol Med*.2018;123(7) : 10.1007/s11547-018-0870-y 524-534 10.1007/s11547-018-0870-y 29508240

[2] Diaz-RuizAOrbe-ArteagaURiosCRoldan-ValadezE: Alternative bibliometrics from the web of knowledge surpasses the impact factor in a 2-year ahead annual citation calculation: Linear mixed-design models' analysis of neuroscience journals. *Neurol India*.66(1) : 10.4103/0028-3886.222880 96-104 10.4103/0028-3886.222880 29322967

[3] Villaseñor-AlmarazMIslas-SerranoJMurataCRoldan-ValadezE: Impact factor correlations with Scimago Journal Rank, Source Normalized Impact per Paper, Eigenfactor Score, and the CiteScore in Radiology, Nuclear Medicine & Medical Imaging journals. *Radiol Med*.2019; 10.1007/s11547-019-00996-z 10.1007/s11547-019-00996-z 30725395
